# Gap analysis for drug development policy-making: An attempt to close the gap between policy and its implementation

**DOI:** 10.1371/journal.pone.0220605

**Published:** 2019-08-06

**Authors:** Ria Christine Siagian, Dumilah Ayuningtyas

**Affiliations:** School of Public Health Universitas Indonesia, Depok, Indonesia; Institute for Advanced Sustainability Studies, GERMANY

## Abstract

**Introduction:**

Most drug development policies in developing countries are enacted without achieving the desired results. This study aims to determine the prioritization of drug development in Indonesia through the evidence-based policymaking process in order to close the distance between stated policy goals and the realization of planned goals.

**Methods:**

A quantitative approach in the form of cross-sectional research using a structured survey was adopted and validated using a set of techniques involved in the calculation of a structural equation model. An independent samples t-test was used to test the significance of the differences between two views: pharmaceutical industries and the government of Indonesia.

**Findings:**

The study reveals that pharmaceutical industries and governments were highly consistent in their perceived challenges in facing the drug development. It also reveals drivers and weaknesses of drug development, including market opportunities, push-pull-regulatory pull factors and regulation, as priorities for improvement.

**Conclusions:**

Gap analysis based on a structural model was borne out to address gap challenges between policy and its implementation, with the use of evidence-based policymaking.

## Introduction

It has been observed that policy implementation is a major problem in developing countries [1,2]. Failures in achieving the desired goals of a policy can be attributed to a mismatch in resources, a lack of communication bridging research to policy, a lack of strategy, governance instability and a lack of political commitment [1–5]. It is worth noting that more empirical evidence in developing countries is required to conduct a successful implementation [3]. Interest in drug development has increased significantly in recent years. Yet, many researches on drug development in developing countries have generated many debates. The World Health Organization reported that several developing countries such as Argentina, Bangladesh, Colombia, China, Ethiopia, India, Thailand, Uganda, Jordan and Indonesia have put in the effort to support drug development by releasing favorable policies. The governments of the aforementioned countries have supported drug development by creating an innovation environment through drug regulations, scientific capabilities, infrastructure, financial support for research and development (R&D), partnerships, intellectual property protection, innovation diffusion and market opportunities [6–9]. Apparently, only China and India have succeeded in occupying a leading position in the pharmaceutical sector [8,9]. Nevertheless, China and India still struggle to develop real innovative medicines due to insufficient manpower for new drug R&D [10,11]. Albeit, majority of developing countries pose similar situation; this paper attempts to find empirical evidence in the Indonesian setting where pharmaceutical industries and government must set the development agenda of tomorrow to meet the diverse and changing needs of drug access. The Indonesian government has provided funding, resource support, tax incentives, promotions and a facilitating roadmap for drug development [12], however, there is only a small number of Indonesian pharmaceutical industries engaged in R&D. They prefer to manufacture products at the stages of formulation and/or packaging [12,13].

Policy implementation is a stage of policymaking process which refers to the actions taken to put the policy into practice. A problem arises when the goals of the policies are not achieved. The government has to overcome gaps and make decisions under the conditions of uncertainty, because decisions are often made according to what is available at the time or are controlled by political factors instead of empirical ones [14]. In politics, perception plays a primary role and changes can be made through negotiations. On the other hand, evidence is not considered primary and tends to be ignored in the policymaking process. Gaps in the implementation of a policy can occur when there is a difference between expectations and the implementation of the policy in question [15]. Strategies must be developed in order to avoid a gap between the expectations of a policy and its actual outcome. During the policymaking process, the government has to interact with a large number of different external sectors, each of which have different interests and needs. It is widely perceived that the government’s ultimate objective is to improve the ability of pharmaceutical industries to conduct drug development [16]. However, this normative view may not always reflect its practice. Government agencies and external actors approach issues from different perspectives. Knowledge sharing can lead to a gap in viewpoints between the government and the external sector which must be overcome. Such strategies need to take into account financial aspects, policy capacities (managerial and technical) and the anticipation of support or resistance from within and outside the government [15]. Minimizing the uncertainties associated with drug development by strengthening the aforementioned factors is a major catalyst that can encourage pharmaceutical industries to invest more money in drug development [14,15,17]. Through evidence-based policy making, it is expected that gaps can be identified [14,17] by analyzing (1) factors outside institutions, (2) time and resources, (3) causal relations, (4) agreement on objectives, (5) communication and coordination [15].

Gap analysis is a method that can be used to examine the similarities and differences between the views of multiple different stakeholders, including pharmaceutical industries and policymakers. Furthermore, it examines their plans, involving pharmaceutical industry capabilities (pharma capabilities) and innovation incentives, to accelerate drug development. This approach can offer empirical insight into the gaps that may arise from the different perceptions on both current and potential situations. Several service quality assessment approaches that define the service quality gap concept were found in the literature. In the health sector, the quality improvement approach is often implemented to measure the quality of healthcare services and patient satisfaction. The measurement can then be used to evaluate current performance and identify areas that require more attention. Importance–performance analysis has been widely used to examine the relationship between importance and performance, which can then be used to set priorities during the policymaking process. This ascertains the assumption that attribute performance has little impact on the determination of the priorities when stated importance is low, meaning that stated importance may be more useful than previously thought. A combination of service quality, importance performance analysis and the structural equation model (SEM) was also found in the literature, using SEM as the method of analysis to produce regression equations for the performance and importance of the gaps [18].

**Fig 1:**
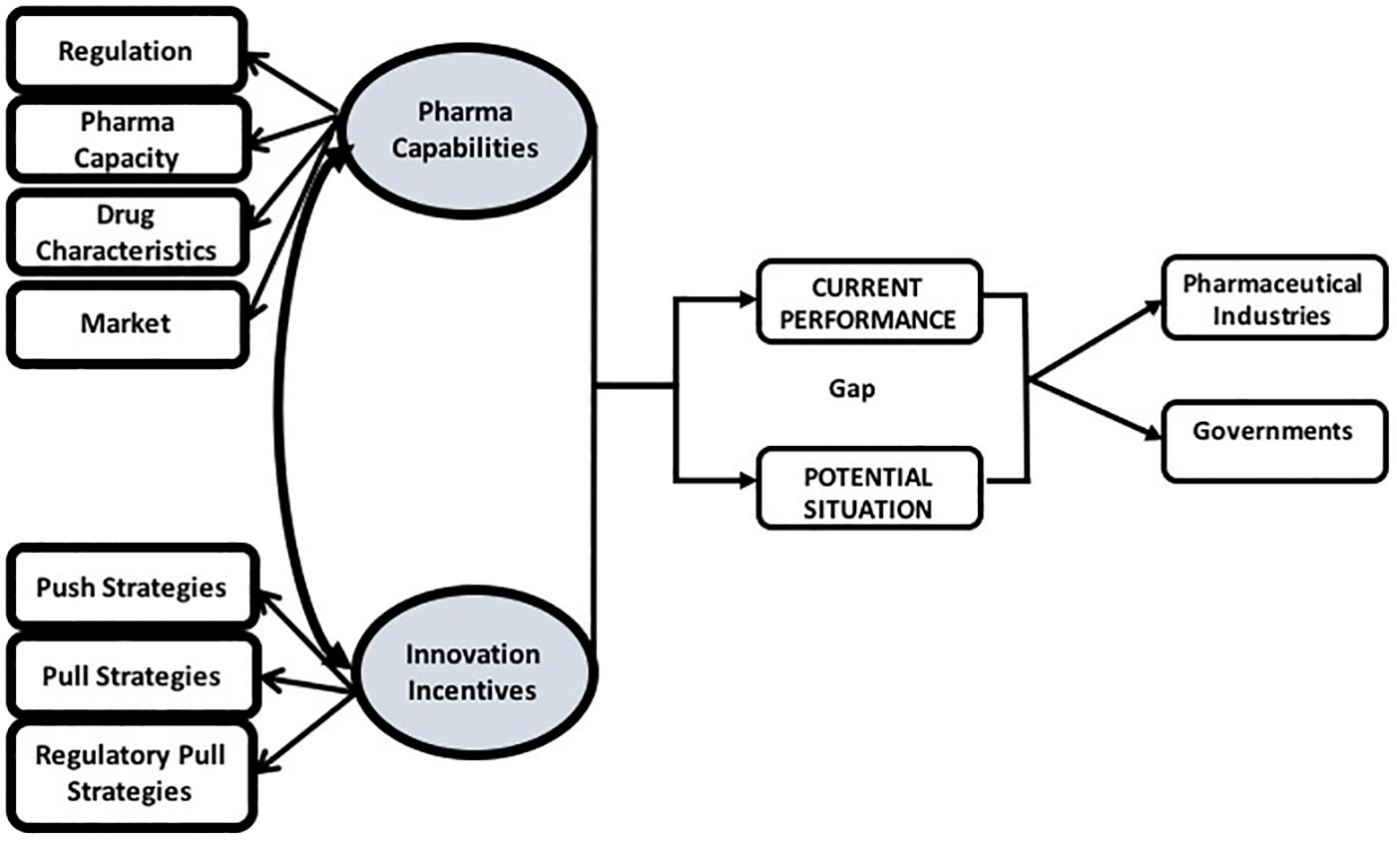
Conceptual model.

Although several studies have extensively examined the lack of drug development in developing countries, very little research has focused on the challenges and opportunities facing the decision to proceed drug development from the perspectives of different stakeholders. Depending on national circumstances, countries have to set their own priorities, according to their pharmaceutical industry capabilities and the innovation incentives they offer. This is worth pursuing if these priorities are assessed from the perspectives of the government and pharmaceutical industries. The analysis can help identify the drivers of and barriers to drug development during policymaking process, and can therefore bridge the gap between policy and its implementation. This study aims to determine the priorities of drug development in Indonesia through the evidence-based policymaking process in order to close the distance between the stated policy goals and the realization of those goals. A conceptual model was developed to identify gaps in the point of views of the pharmaceutical industry and the government. It is composed of constructs, namely pharma capabilities and innovation incentives, which involve seven causal relationships of drug development elements derived from the literature. They include Regulation, Pharmaceutical Industry Capacity (Pharma Capacity), Drug Characteristics, Market Opportunities (Market), Push Strategies, Pull Strategies and Regulatory-Pull Strategies (Fig 1) [19–28]. The model assumes that pharma capabilities have a positive impact on innovation incentives, and vice versa.

## Materials and methods

This study was approved by the Ethics Committee School of Public Health Universitas Indonesia, approval number 662/UN2.F10/PPM.00.02/2018 dated 7^th^ August 2018.

### Study design and sampling methods

In order to generate the dataset, an electronic survey was conducted among the top level of pharmaceutical industries and drug-development related governments in Indonesia. A focused sample procedure was conducted. Governments in this study were restricted to the institutions stated on the presidential instruction on facilitating drug development in Indonesia, while all pharmaceutical industries in Indonesia were included. A total of 200 questionnaires were electronically distributed to the top-level pharmaceutical industries and relevant government institutions, identified by the database of Indonesian Drug Regulatory Authority (DRA).

### Variables of the questionnaire

The questionnaire used for this study was developed by revising and complementing questionnaires of other studies, based on in-depth interviews with 6 (six) multi-stakeholders involved in drug development field, representing governments and pharmaceutical industries [19–22,27–31]. The questionnaire was composed of 47 questions on pharma capabilities and 19 questions on innovation incentives. Each item was scored on a 5-point Likert scale, with the measurement of potential situation ranging from 1 (Not at all potential) to 5 (extremely potential), and the measurement of performance ranging from 1 (strongly disagree) to 5 (strongly agree). Table 1 shows the measures and items associated with the constructs.

**Table 1:** Operationalization of the constructs.

Construct	Measure	Item	CURRENT PERFORMANCE		POTENTIAL SITUATION	
			Loadings	CR	Loadings	CR
Pharma Capabilities	Regulation	Drug development	0.7	0.9	0.7	0.9
		Drug registration	0.7		0.7	
		National essential medicines	0.7		0.5	
		Drug pricing	0.6		0.5	
		Investment	0.8		0.6	
		National components	0.7		0.6	
		Regional harmonization	0.8		0.7	
		Global policy	0.8		0.7	
	Pharma Capacity	Competent human resources	0.8	0.9	0.8	1.0
		Innovative management	0.8		0.8	
		Good Manufacturing Practice (GMP) certified facilities	0.7		0.8	
		Quality testing facilities	0.8		0.9	
		Calibrated equipment	0.8		0.9	
		Validated analysis methods	0.8		0.9	
		Ability to proceed R&D on drug discovery	0.7		0.7	
		Ability to proceed R&D on raw material process	0.6		0.7	
		Ability to proceed R&D on bulk process or formulation	0.6		0.7	
		Ability to proceed R&D on filling	0.5		0.7	
		Drug development program for efficiency	0.7		0.7	
		Partnership between pharmaceutical industries	0.7		0.7	
		Public Private Partnership	0.6		0.7	
		Partnership with international network	0.5		0.7	
		Access to high quality of raw material and packaging	0.7		0.7	
	Drug Characteristics	Non-clinical trial and phase 1,2,3 of clinical trials	0.9	0.9	0.8	0.9
		Bioequivalence / bioavailability for efficacy	0.9		0.9	
		Bioequivalence / bioavailability for safety	0.8		0.8	
		Availability of infrastructure and human resources for clinical trial	0.5		0.5	
	Market	Affordable research-based drug	0.6	0.9	0.5	0.9
		Return of investment for biopharmaceuticals	0.8		0.8	
		Return of investment for herbal medicines	0.6		0.7	
		Return of investment for raw materials	0.6		0.7	
		National market size	0.6		0.7	
		Global market size	0.6		0.7	
		Available government program	0.7		0.7	
		Information on Procurement	0.7		0.7	
		Global determination of specific drug	0.8		0.7	
		Private sector engagement	0.6		0.7	
		E-Purchasing system	0.7		0.5	
		Promotion	0.5		0.4	
Innovation Incentive	Push Strategies	Funding for R&D	1.0	0.9	0.9	0.9
		Funding for clinical development program	1.0		0.9	
		Tax holiday and tax allowance	0.9		0.8	
		Government investment on infrastructures	0.8		0.7	
		Long-term collaboration	0.8		0.7	
	Pull Strategies	Intellectual property protection	0.7	0.9	0.8	0.9
		Diffusion of products	0.8		0.7	
		Advanced purchase commitment	0.9		0	
		Market exclusivity	0.9		0.8	
		Reward	0.9		0.8	
	Regulatory Pull Strategies	Shortened registration process	0.9	1.0	0.9	1.0
		Simplification of procedures	1.0		1.0	
		Adaptive regulation	1.0		0.9	

### Data analysis

One hundred and twenty-five (125) questionnaires were collected (approximately 63%), which were used in the analysis. The conceptual model was tested applying the covariance-based SEM program AMOS 24.

## Results

### Assessing measurement model and structural model validity

Based on the existing literature, a conceptual model was developed and tested using the data and information gathered via the questionnaire covering the main elements of drug development. The structural equation modeling empirically tested and validated the proposed causal relationships between the variables concurrently. The objective of analyzing the measurement model is to evaluate the model’s validity and reliability, whereas the objective of analyzing the structural model is to determine the significance of the relationships between the independent and dependent constructs [32]. The convergent validity (standardized loading estimates) and construct reliability (CR) were analyzed to test the reflective measurement models, and the results are shown in Table 1. The standardized factor loadings for all reflective items lie well with a value of 0.5 or higher. The CR, a measure of reliability and internal consistency, ranges from 0.9 to 1.0, ensuring a high level of reliability [33]. The CR replaces the internal reliability measurement using Cronbach’s Alpha since this study was analyzed using SEM. Hair et al. (2014) agreed that the values of CR should be 0.7 or higher to ensure adequate convergence or internal consistency. The latent construct is considered valid when the fitness indexes achieve the model fit categories. The fitness indexes, as the constructs in final models, had achieved the required level as summarized in Table 2 [33,34]. The results indicated the well-fitting models, confirming whether a theoretical measurement model is valid.

**Table 2:** Fit Index of SEM calculation.

Index		Acceptable threshold level [33,34]	Fitted Structural Model	
			Current Performance	Potential Situation
Normed Chi-squared	$\frac{x^{2}}{\left(df\right)}$	$1<\frac{x^{2}}{\left(df\right)}<3$	1.79	1.80
Root Mean Squared Error of Approximation	RMSEA	≤ 0.08	0.08	0.08
Root Mean Square Residual	RMR	≤0.10	0.12	0.09
Tucker Lewis Index	TLI	≥0.90 Good Fit 0.8≤ TLI,CFI ≤0.9 marginal fit (0 = poor, 1 = perfect)	0.81	0.80
Comparative Fit Index	CFI		0.82	0.81

**Fig 2:**
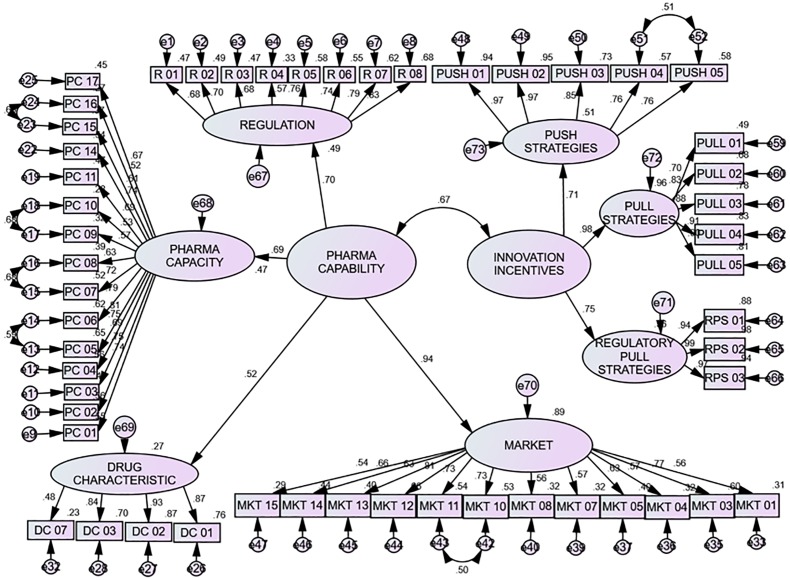
Final performance model.

The performance model given in the Fig 2 shows that pull strategies and market have the biggest impact to innovation incentives and pharma capabilities, respectively. Whereas five other elements are somewhat less where drug characteristics have the smallest impact. The factors contributing to the current performance of drug development in the order of priority are pull strategies, market, regulatory pull strategies, push strategies, regulation, pharma capacity, and drug characteristics. Their regression weights/factor loadings are 0.98, 0.94, 0.75, 0.71, 0.70, 0.69, 0.52, respectively. The loadings are used as regression coefficients to estimate of the values of any construct in the model:

$$\begin{array}{l} Current\;performance\\ =0.98\;Pull\;strategies+0.94\;Market+0.75\;Regulatory\;pull\;strategies\\ +0.71\;Push\;strategies+0.70\;Regulation+0.69\;Pharma\;capacity\\ +0.52\;Drug\;characteristics \end{array}$$

The squared multiple correlations (R^2^ values) indicate that pull strategies, market, regulatory pull strategies, push strategies, regulation, pharma capacity, and drug characteristics with values 0.96, 0.89, 0.56, 0.51, 0.49, 0.47 and 0.27 respectively, predict the current performance of drug development (Table 3).

**Table 3:** The percentages of explained variance (R^2^ values).

Current Performance	Potential Situation
Pharma capabilities are reflected by: Regulation 49% Pharma capacity 47% Drug Characteristics 27% Market 89%	Pharma capabilities are reflected by: Regulation 50% Pharma capacity 57% Drug Characteristics 32% Market 71%
Innovation incentives are reflected by Push strategies 51% Pull strategies 96% Regulatory pull strategies 56%	Innovation incentives are reflected by Push strategies 60% Pull strategies 92% Regulatory pull strategies 61%

**Fig 3:**
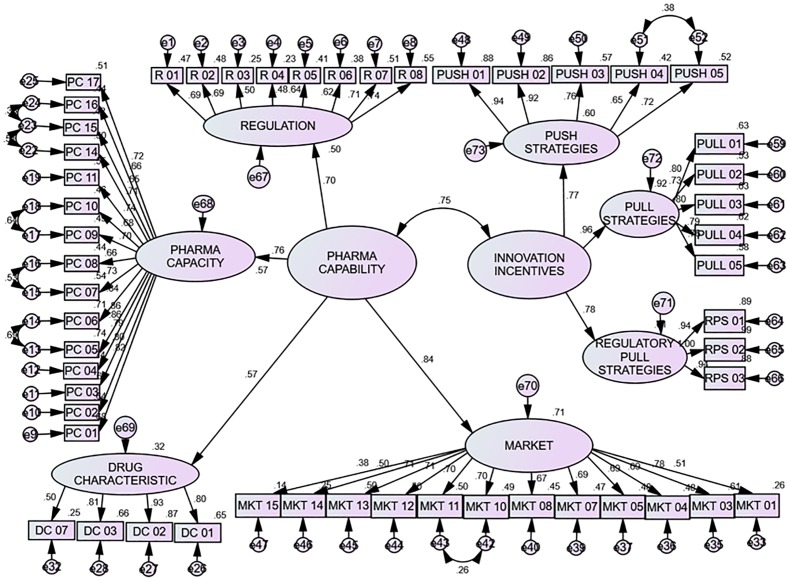
Final potential situation model.

The potential model shown in Fig 3 shows a similar pattern, in which pull strategies and market potentially have the biggest potential impact on drug development, followed by regulatory pull strategies, push strategies, pharma capacity, regulation and drug characteristics. Their estimated coefficients are 0.96, 0.84, 0.78, 0.77, 0.76, 0.70, 0.57, respectively. Thus, for any particular values for the variables, an estimated value for the potential situation can be obtained:

$$\begin{array}{l} Potential\;situation\\ =0.96\;Pull\;strategies+0.84\;Market+0.78\;Regulatory\;pull\;strategies\\ +0.77\;Push\;strategies+0.76\;Pharma\;capacity+0.70\;Regulation\\ +0.57\;Drug\;characteristics \end{array}$$

The squared multiple correlations (R^2^) indicate that pull strategies, market, regulatory pull strategies, push strategies, pharma capacity, regulation, and drug characteristics, with a value of 0.92, 0.71, 0.61, 0.60, 0.57, 0.50 and 0.32 respectively, predict the potential drug development (Table 3).

### Gap analysis

**Fig 4:**
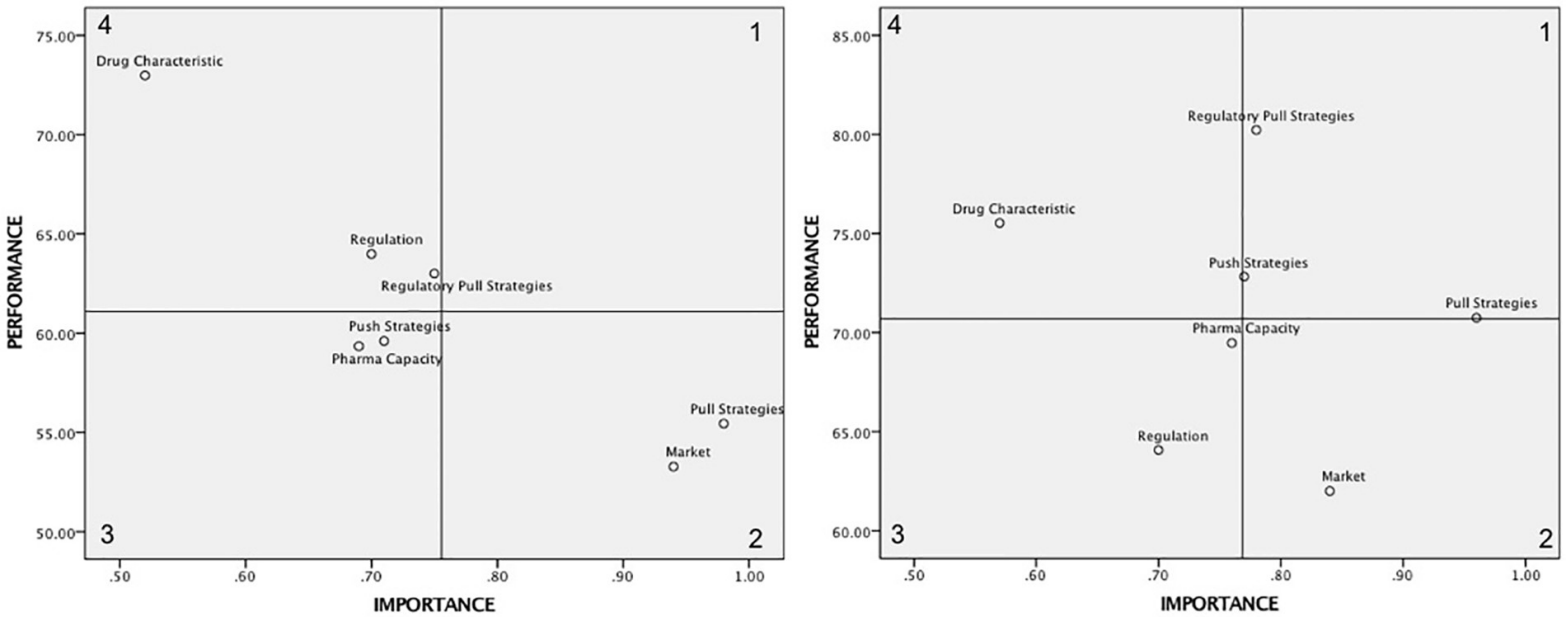
Importance—Performance map for current performance (left) and potential situation (right).

An importance performance analysis was run to illustrate the diagnostic value of the above model. The scheme is characterized by a four-quadrant graph where each one has a meaning for future action. The importance-performance can help identify policies that need to be prioritized according to their level of importance. More specifically, high importance that requires performance improvements will be prioritized [35,36]. The importance–performance analysis focuses on the importance of the seven drivers of drug development in current performance, as depicted in Fig 2, and in potential situation as depicted in Fig 3. In the SEM, the analysis estimates the relationships in the model (the importance of each latent variable) and the values of the latent variables (performance) [18]. Ringle and Sarstedt (2016) explained the computation of the importance and performance values. The importance values emerge from the total effects of the estimated relationships, whereas the performance values are determined by rescaling the indicators Likert scores to a range from 0 to 100, indicating the higher the score, the better the performance. The result of this computation is summarized in Table 4, and was used to create the importance-performance map as shown in Fig 4.

Performance=∑Rescaledlatentvariablescoresξ (1)

$$Rescaled\;latent\;variable\;scores\;\xi_{i}=\{\frac{\left(Score\;\xi_{i}-min\xi_{i}\right)}{\left(max\;\xi_{i}-min\;\xi_{i}\right)}*100\}*Rescaled\;weights$$ (2)

$$Rescaled\;weights=\frac{\;Standardized\;loading\;estimates\;\lambda_{i}/SD\;indicator\;\lambda_{i}}{\sum_{\lambda_{i-n}}\left(Standardized\;loading\;estimates/SD\right)}$$ (3)

*i* = 1, …5; *n* = 1, …, 125.

**Table 4:** Values for importance performance map.

STRATEGY	CURRENT PERFORMANCE		POTENTIAL SITUATION	
	IMPORTANCE	PERFORMANCE	IMPORTANCE	PERFORMANCE
Regulation	0.70	63.98	0.70	64.07
Pharma Capacity	0.69	59.34	0.76	69.47
Drug Characteristics	0.52	72.98	0.57	75.53
Market	0.94	53.27	0.84	62.00
Push Strategies	0.71	59.60	0.77	72.82
Pull Strategies	0.98	55.44	0.96	70.74
Regulatory Pull Strategies	0.75	63.00	0.78	80.22

In terms of setting priorities to the policymaking process of drug development (Fig 4), the seventh strategies need improvement due to no element plotted in quadrant 1 (high importance/high performance). This indicates no major strength of any element in current situation. Pull and market strategies that fall in the top priority quadrant 2 (high importance/low performance) require immediate corrective action to enhance their performances. Although they were predicted to make the greatest impact on drug development with the importance value of over 0.9, they were given less attention compared to the other five strategies represented by their lowest performance ratings. The push and pharma capacity strategies are located in the low priority quadrant 3 (low importance/low performance) representing elements with no contribution to drug development. The importance of drug characteristics, regulation and regulatory-pull strategies were rated low with relatively high performance, located in quadrant 4 (low importance/high performance). These elements were also perceived of no impact on drug development. In comparison with the other strategies and when policymakers aim at increasing the performance, the pull and market strategies should be put in first priority. Market, pharma capacity and drug characteristic strategies potentially fall in the same quadrant while regulation is moved to quadrant 3. It is still prioritized although less important than the aforementioned strategies. It was perceived that all innovation incentives comprising push–pull–regulatory pull strategies would be potentially contained in quadrant 1 (Fig 4 right). This suggests that putting them into top priority offer potential major improvement in terms of the potential situation level.

### Perception comparisons of performance and potential between multi-stakeholders in drug development

The t-test was used straightforward to compare the perspectives of multi-stakeholders on the elements needed to drive drug development. The results are displayed in Table 5. They were highly consistent in their perspectives on both the current and potential situations of drug development. Leaders in both the government and pharmaceutical industries agreed that all elements are drivers for drug development. However, the governments had a stronger opinion about regulation and market than the pharmaceutical industries. It should be noted that the government is perceived to be more likely to influence the decision-making process involved in drug development. Given this finding, market opportunities and regulation should be placed into priority, as the government has the capacity to intervene those two implementations.

**Table 5:** t Tests of responses.

Item	The current performance				The potential situation			
	Government Mean	Pharmaceutical Industry Mean	t	P value	Government Mean	Pharmaceutical Industry Mean	t	P value
Regulation	3.68	3.20	2.229	0.028	4.00	3.46	2.883	0.005
Pharma capacity	3.59	3.33	1.339	0.183	3.85	3.75	0.512	.609
Drug Characteristics	3.98	3.78	0.936	0.351	4.07	3.94	0.653	0.515
Market	3.56	3.05	2.833	0.005	4.02	3.39	3.546	0.001
Push Strategies	3.51	3.37	0.497	0.620	4.16	3.88	1.260	0.210
Pull Strategies	3.56	3.18	1.308	0.193	4.11	3.80	1.394	0.166
Regulatory Pull Strategies	3.79	3.49	0.919	0.360	4.26	4.20	0.241	0.810

## Discussion

This study examined the interactions between the key elements of regulation, pharmaceutical industry capacity, drug characteristics, market opportunities, and push–pull -regulatory pull factors, and their impact on pharmaceutical industry capabilities and innovation incentives with respect to strategies for drug development in Indonesia. Some key findings are worthy of discussion. First, the statistical results confirm a strong relationship between each element and the two dimensions of drug development (pharmaceutical industry capabilities and innovation incentives), implying that an optimal outcome can only be achieved through the rigorous implementation of approaches. Subsequently, given the contribution of those seven elements to drug development, this study confirms the importance of the Indonesian government’s role in prioritizing innovation incentives and market to encourage drug development. They consistently presented the strongest causal relationship in current situation and in the future. Thus, the findings provide further insights for policy-making process. There are challenges for policymakers to prioritize weak strategies in order to enhance their qualities for competitive advantages. No element located in the “major strength” area implies that drug development is not attractive in current situation but may be highly attractive in the future. To remain focused on the goal of having local drug development, thus, the policymakers need to enhance the quality of pull and market strategies. Speed to market can benefit from government policy support, in which delays in market access due to regulatory environment can lead to a cost of delay [37]. Universal health coverage in Indonesia should have directly impacted the pharmaceutical sector since it was implemented in 2014. However, due to the system prioritizing low prices instead of local production, drug development is not yet attractive [12,38]. On another note, the findings of this study are consistent with those conducted in India and China, where government intervention through innovation incentives and market strategies were found to be directly linked to drug development [8,9]. Extensive studies support the government’s offers for innovation incentives to boost drug development, and a lack of this intervention on several drugs which may not yield a high return of investment is clearly impacting the access to the drug [20,27].

Another finding is that the performance of regulation can potentially drop, in contrast to other relatively sustainable elements. The performance of drug characteristics and regulation strategies exceed that of the other five elements, but a relatively low (below average) importance. The placement of these elements in the quadrant 4 may imply that these strategies are not part of the overall business strategy, giving no impact to the decision to pursue drug development. Drug development will be influenced less by performance when the importance of these elements is low. One possible explanation for this finding could be associated with the regulatory pressures in Indonesia, driving pharmaceutical industries to be more focused on regulatory requirements of drug characteristics (safety, efficacy and quality). This suggests that regulation should be put into the strategies in order to avoid potentially lower performance if it is abandoned. Pharmaceutical industries and the government should be aware that the regulation is very dynamic and the complexity of a new drug will require more assessments. They have to pay more attention to the implementation of regulation and to become more knowledgeable on regulation to prevent this element’s performance from dropping.

It must be noted that the government and pharmaceutical industries had different perspectives on regulation and market. The available literature attributed the difference to the government’s concern for communities, in contrast to the market-oriented focus of pharmaceutical industries [16,26]. In a field like pharmaceuticals, where mistakes can have major consequences, the safety, efficacy and quality of pharmaceutical products are primary reasons for incorporating human factors. The government, whose perspectives are stronger than that of pharmaceutical industries, should strengthen the country’s regulatory system by improving its knowledge on the various types of drug development, as well as encouraging pharmaceutical industries to better understand regulatory requirements (regulatory intelligence) in order to produce high-quality pharmaceutical drugs and increase the competitiveness of high-value-added pharmaceutical products. Roles of the government’s involvement in evidence-based policy implementation found in this study concur with the argument that governments strongly influence on a spectrum of policies from voluntary to mandatory [39].

The strength of this study lies in its exploration of multi-stakeholders’ perspectives in pursuing drug development, top levels of governments and pharmaceutical industries. However, the scope of this study did not include an examination of how political factors (players, perception, power and position) or political stability (a country’s governance) affect drug development, which should be researched in future studies.

## Conclusion

While it is easy to appreciate the importance of drug development, making it an integral part of a business practice is not a simple task. Encouraging pharmaceutical industries in developing countries to pursue drug development remains a challenge. Drug development in developing countries requires the exploration of novel approaches, some of which may fail. Early research was empirically driven but often did not pay attention to the theoretical underpinnings of policy implementation in practice. This study clarified the importance of integrating policy, research and policy implementation, by identifying the problems in drug development that need to be prioritized. A gap analysis based on a structural model may serve as an important tool to identify strengths and weaknesses, and it may be useful in evaluating health-related policies. A four-quadrant matrix helps establish and define an action plan to minimize the differences between the current performance and potential situation when the policy is implemented. The study found that innovation incentives and market opportunities were excellent strategies supporting drug development. Market opportunities require the most attention, whereas regulation is another strategy that cannot be abandoned.
